# Detection of increased coronary microvascular permeability with MRI T1 mapping and gadolinium-labeled albumin

**DOI:** 10.1186/1532-429X-18-S1-W3

**Published:** 2016-01-27

**Authors:** Sophia X Cui, Brent A French, Frederick H Epstein

**Affiliations:** 1 grid.27755.32000000009136933XBiomedical Engineering, University of Virginia, Charlottesville, VA USA; 2 grid.27755.32000000009136933XRadiology, University of Virginia, Charlottesville, VA USA

## Background

Conditions such as obesity and diabetes lead to coronary microvascular disease and, subsequently, an increased risk of adverse cardiac events. Increased microvascular permeability is thought to be an early biomarker of coronary microvascular disease. While prior studies have used MRI with gadolinium-labeled albumin, a macromolecular contrast agent, to detect myocardial infarction, we hypothesized that MRI T1 mapping with gadolinium-labeled albumin could detect more subtle changes in coronary microvascular permeability such as those induced by noninjurious pharmacological methods.

## Methods

Wild type male C57Bl/6 mice (n = 7) were imaged at 7T. Mice were anesthetized with 1.25% isoflurane and maintained at 36 ± 0.5°C during MRI. T1 mapping was performed at baseline and at 7, 14, 21, and 28 minutes after administering gadolinium-labeled albumin (4 mL/kg; Galbumin, BioPal, Worcester, MA) and, in a separate session, at the same times without administering contrast agent. Control studies without pharmacologically increasing permeability and studies where LNAME (2 mg/kg) and endothelin-1 (ET-1) (1 nmol/kg) were injected 3 and 10 minutes after baseline imaging to increase coronary microvascular permeability [1] were performed. Compressed-sensing accelerated (rate 2) spiral Look-Locker imaging was used for T1 mapping. The partition coefficient, defined as the ratio of contrast agent concentration in the myocardium to that in the blood, was used to quantify microvascular permeability.

## Results

**Figure 1:**
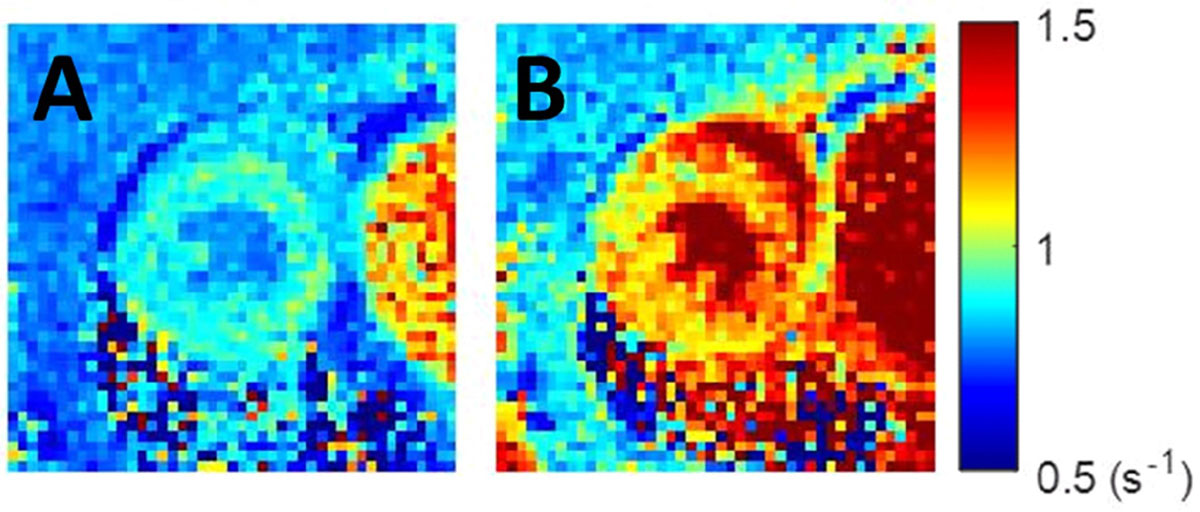
**Example R1 maps of the mouse heart before (A) and 14 minute after iv injection of 4 mL/kg Gd-labeled albumin (B)**.

**Figure 2:**
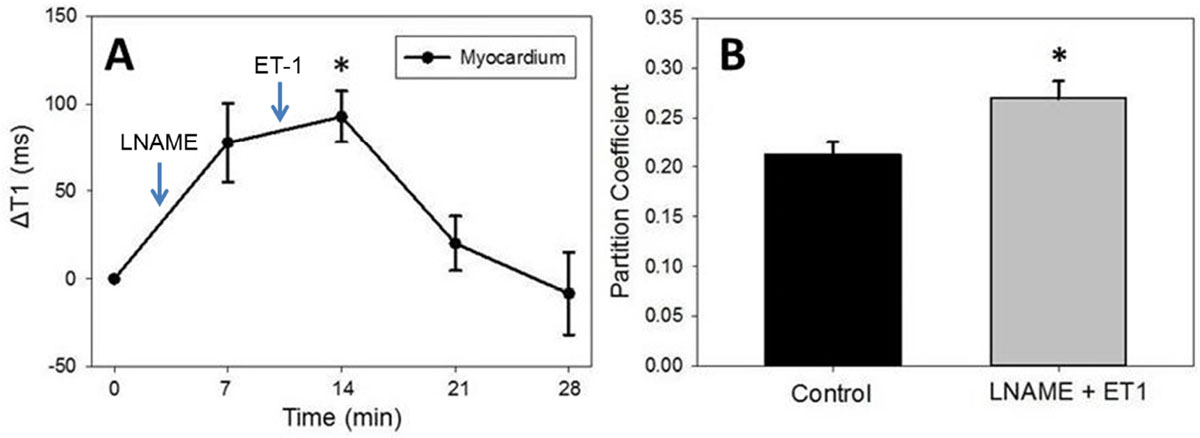
**(A) Native myocardial T1 values increase and return to baseline after iv injection of LNAME and ET-1**. At the peak of the response (14 minutes), the injection of LNAME and ET-1 caused an average of 92 ms increase in native myocardial T1 (*p < 0.05 vs.baseline). (B) Without pharmacological agents, the partition coefficient of gadolinium-labeled albumin was 0.21 ± 0.017 and it increased to 0.27 ± 0.013 after administering LNAME and ET-1 (*p < 0.05 vs. control). All data shown are mean ± standard error.

Figure 1 shows example R1 (1/T1) maps acquired before and 14 minutes after injection of gadolinium-labeled albumin. As shown in Figure 2, T1 mapping after the injection of LNAME and ET-1 detected a transient increase in native T1 due to edema (p < 0.05). T1 mapping before and after injecting gadolinium-labeled albumin detected a significant increase in the partition coefficient after administration of pharmacological agents compared to control (0.27 ± 0.017 LNAME and ET-1 vs 0.21 ± 0.013 control; p < 0.05), reflecting detection of the induced increase in microvascular permeability.

## Conclusions

T1 mapping with gadolinium-labeled albumin can detect pharmacologically-induced changes in coronary microvascular permeability. These methods hold potential for the serial noninvasive assessment of the coronary microvasculature in preclinical models of heart disease and for studies evaluating potentially protective therapies for the microvasculature. Future work will investigate the use of clinically-applicable agents such as albumin-binding gadolinium (Ablavar, Lantheus, MA) as a means to potentially translate these methods for studies of coronary microvascular disease in human patients.

## References

[1] Filep J (1993). BJP.

